# Disposable Electrochemical Aptasensor Based on Graphene Oxide-DNA Complex as Signal Amplifier towards Ultrasensitive Detection of Ochratoxin A

**DOI:** 10.3390/mi13060834

**Published:** 2022-05-26

**Authors:** Yang Hu, Hanyin Xie, Jiaying Hu, Danting Yang

**Affiliations:** Department of Preventative Medicine, Zhejiang Key Laboratory of Pathophysiology, School of Medicine, Ningbo University, 818 Fenghua Road, Ningbo 315211, China; 2011101047@nbu.edu.cn (Y.H.); 196002099@nbu.edu.cn (H.X.); 196002028@nbu.edu.cn (J.H.)

**Keywords:** aptasensor, graphene oxide, reduced graphene oxide/AuNPs, ochratoxin A, signal amplification

## Abstract

Signal amplification is crucial in developing a reliable disposable screen-printed carbon electrodes (SPCEs)-based biosensor for analyte detection with a narrow detection window. This work demonstrated a novel label-free electrochemical aptasensor based on SPCEs for the ultrasensitive detection of ochratoxin A (OTA). The graphene oxide-DNA (GO-DNA) complex as a signal amplifier with easy preparation was investigated for the first time. The proposed aptasensor based on the SPCEs/GO/cDNA-aptamer/3D-rGO-AuNPs structure was formed through the hybridization of aptamer-linked 3D-rGO/AuNPs and its complementary DNA-linked GO (GO-cDNA). The presence of OTA was discerned by its specific aptamer forming a curled OTA-aptamer complex and releasing the GO-cDNA from the surface of SPCEs. The resulting OTA-aptamer complex hindered interfacial electron transfer on the sensing surface, leading to the decreased peak current. The GO-cDNA further amplified the peak current change. This electrochemical aptasensor showed a low limit of detection of 5 fg/mL as well as good reproducibility with the relative standard deviation (RSD) of 4.38%. Moreover, the detection result of OTA in the rice and oat samples was comparable with that of the enzyme-linked immunosorbent assay (ELISA) kit. In general, the OTA aptasensor used in this work with convenient preparation, low-cost, good selectivity, high sensitivity and acceptable reproducibility can be proposed as a reliable point-of-care (POC) technique for OTA determination.

## 1. Introduction

Ochratoxin A (OTA) is a ubiquitous mycotoxin produced by *Penicillium* and *Aspergillus* in their secondary metabolism [1], which can be found in a variety of crops such as wheat, corn, or oats [2]. As it can cause varying degrees of damage to human and animals such as nephrotoxicity (Balkan endemic nephropathy, BEN), hepatotoxicity, neurotoxicity, immune-toxicity and teratogenicity [3], OTA has been classified by International Agency for Research on Cancer (IARC) in group 2B of possible human carcinogens [3,4]. Thus, a rapid and sensitive detection strategy is important for the accurate diagnosis of OTA, especially in very low concentration samples.

Currently, the most commonly used methods are the enzyme-linked immunosorbent assay (ELISA) and high-performance liquid chromatography (HPLC) [5,6], which possess high sensitivity and accuracy. However, they require expensive reagents or equipment, and highly trained operators, limiting their wide application in point-of-care (POC) testing, especially in less developed or remote areas. POC biosensing methods with affordable, user-friendly, and disposable devices suitable for on-site measurements are in critical demand. Aptamer-based electrochemical biosensors with high specificity and affinity, fast response, simple operation and low cost are able to overcome limitations of immuno-based electrochemical sensors in terms of cross-reactivity, and false screening results, attracting great attention as a reliable and fast POCT in various fields [7,8,9,10]. Specific electrochemical electrodes such as glassy carbon electrodes (GCEs) [11,12], carbon paste electrodes (CPEs) or graphite pencil electrodes [13,14] have been used to develop the electrochemical aptasensor of OTA detection. For instance, our previous work demonstrated a label-free aptasensing device based on 3D-rGO/AuNPs-modified GCEs to achieve the sensitive and selective determination of OTA [15]. The aptasensor exhibits an ultrasensitive limit of detection (LOD) of 0.34 pg/mL with good reproducibility. However, the above solid electrodes may suffer cross-contamination, surface poisoning or time-consuming surface cleaning, which are not suitable for single-use disposable systems [16]. Disposable screen-printed carbon electrodes (SPCEs) which are regarded as low cost, have easy miniaturization and great potential for mass production, are more favorable in POC [17,18].

To our knowledge, there are only a few articles focused on the design of disposable and portable electrochemical aptasensors for the detection of OTA [19,20]. For instance, Zejli et al. reported an aptasensor based on polythiophene-3-carboxylic acid modified SPCEs for OTA detection with an LOD of 0.125 ng/mL [19]. However, the LOD is very poor and the linear range is relatively narrow. In order to address the issue, efforts have focused on the use of signal amplifiers such as an enzyme [21,22] or multiple DNA hybridization complex [23]. For instance, with the introduction of exonuclease and β-cyclodextrin as signal amplifiers in SPCEs, the LOD of OTA using SPCEs-based aptasensor can be reached at as low as 3 pg/mL [20]. However, the enzyme-based method is more susceptible to temperature and pH as well as being costly, while the multiple DNA hybridization-based signal amplification cycle is very complicated. Therefore, the development of an enzyme-free aptasensor with low cost, high stability, and easy preparation is of great importance. Graphene oxide (GO) has been reported with various oxygen functional groups (hydroxyl, carboxyl and epoxy functional groups) on the basal plane as well as at the edges of the GO nanosheet [24], which is easily modified with biomolecules such as DNA [25]. In addition, GO is regarded as an excellent fabrication nanomaterial of electrochemical biosensors due to its ability to promote electron transfer and low cost [26]. Inspired by these, we designed a novel label-free aptasensor using aptamer linked 3D-rGO/Au NPs nanocomposites as a disposable SPCEs substrate and GO-cDNA complex as a signal amplifier to realize OTA ultrasensitive determination. The proposed OTA aptasensor showed simple preparation, an ultrasensitive detection limit, good reproducibility, and high specificity. In addition, the detection results of rice and oat samples can be compared with commercial ELISA results, indicating its potential use in POC, especially for resource-limited settings.

## 2. Experimental Section

### 2.1. Chemicals and Materials

Methanol, potassium chloride (KCl), potassium ferricyanide (K_3_[Fe(CN)_6_]), potassium ferrocyanide (K_4_[Fe(CN)_6_]), glucose (C_6_H_12_O_6_), disodium hydrogen Phosphate dodecahydrate (Na_2_HPO_4_·12H_2_O), disodium hydrogen phosphate dihydrate (NaH_2_PO_4_·2H_2_O), Tris(2-carboxyethyl) phosphine(TCEP), chloroauric acid (HAuCl_4_·4H_2_O), 1-ethyl-3-(3-(dimethylamin)propyl)carbod-iimide hydrochloride (EDC), N-hydroxysuccinimide (NHS), ochratoxin B (OTB), deoxynivalenol (DON), and zearalenone (ZEA), bovine serum albumin (BSA) were purchased from Sigma-Aldrich (Shanghai, China). Tris(hydroxymethyl)aminomethane hydrochloride (tris-HCl), ethylenediaminetetraacetic acid disodium salt (EDTA), and hydrogen peroxide were purchased from Sinopharm Chemical Reagent Co., Ltd. (Shanghai, China). Graphene oxide nanosheets (XF002-1, 500 nm–5 μm; ~99%, Hummers) were purchased from Nanjing XFNANO Materials Tech Co., Ltd. (Nanjing, China). Ochratoxin A was purchased from J&K Scientific Ltd. (Shanghai, China). The OTA binding aptamer with a sequence of 5′-GAT CGG GTG TGG GTG GCG TAA AGG GAG CAT CGG ACA-(CH_2_)_6_-SH-3′ and its complementary DNA (cDNA) with a sequence of 5′-CCT TTA CGC CAC CCA CAC CCG ATC-(CH_2_)_6_-NH_2_-3′ were purchased from Sangon Biotech (Shanghai, China). The commercial ELISA kit was purchased from Multisciences biotech Co., Ltd. (Hangzhou, China). Screen-printed carbon electrodes (SPCEs; DRP-C110) were purchased from Metrohm DropSens (The Swiss). All chemicals were of analytical grade and required no purification. Distilled water (18.2 MΩ·cm^−1^) was used to prepare all aqueous solutions. Rice and oats samples were provided as a gift from Prof. Xing Liu in Hainan University.

### 2.2. Synthesis of 3D-rGO/AuNPs Nanocomposites

The 3D-rGO/AuNPs nanocomposites were synthesized through a one-pot hydrothermal reduction process according to our previous article [15]. Briefly, 20 mg of GO nanosheets were dissolved in 10 mL of water under ultrasonic treatment for 2 h to form 2 mg/mL of GO suspension. Then, 10 mL glucose (2 mg/mL) was added to the above solution followed by the addition of 400 µL of HAuCl_4_·4H_2_O (2%, *w*/*w*) in an ultrasonic bath for 1 h. Afterwards, the homogenous solution was dropped into a Teflon-lined autoclave and reacted in an oven at 180 °C for a full 12 h. After cooling to room temperature, the 3D-rGO/AuNPs hydrogel was washed with distilled water several times and dried with filter paper. Lastly, the 3D-rGO/AuNPs hydrogel was freeze-dried at −50 °C for 24 h and then 3D-rGO/AuNPs nano-powder was obtained for further use.

### 2.3. Preparation of Modified Electrodes

#### 2.3.1. Activation of SPCEs

Firstly, the bare SPCEs were activated under 12 repetitive cyclic voltammetries at the 10 mV·s^−1^ scan rate between 1.0 V and −1.0 V in the 0.01 M H_2_O_2_ (in 0.1 M phosphate-buffered solution, pH 7) [27]. After activation, the electrodes were rinsed with distilled water and dried in air for further experimentation. Activated SPCEs were referred to as aSPCEs for short.

#### 2.3.2. Fabrication of 3D-rGO/AuNPs

The dry 3D-rGO/AuNPs nanocomposites were dissolved in distilled water under ultrasound into different concentrations (0.25, 0.5, 1, and 2 mg/mL), respectively. Then, 10 μL 3D-rGO/AuNPs dispersions were drop-casted on the working electrode surface and left to dry for 2 h at room temperature. Subsequently, the electrode was washed with the 0.01 M phosphate-buffered saline (PBS, pH 7.4) solution three times to remove unbound 3D-rGO/AuNPs and dried in the air for further experimentation.

#### 2.3.3. Fabrication of 3D-rGO/AuNPs/Aptamer

The thiolate-OTA aptamer was activated through incubation with 10 mg/mL TCEP at 37 °C for 1 h. Then, 10 μL of the activated aptamer solution (0.1, 0.5, 1.0, and 2 μM) was dropped on the 3D-rGO/Au NPs-modified aSPCEs working electrode and placed in a 4 °C refrigerator overnight, respectively. Afterwards, the unbound aptamer was removed by careful washing with 0.01 M PBS solution. A total of 10 μL of 10 mg/mL BSA was then dropped on the aSPCEs for 1h to block nonspecific binding sites followed by washing with PBS solution and dried for later use.

#### 2.3.4. Fabrication of 3D-rGO/AuNPs/Aptamer-cDNA/GO

The 100 μL GO solution (0.25, 0.5, 1, and 2 mg/mL) was pretreated with the 100 μL EDC/NHS (37.5 mg/mL/10.5 mg/mL) mixture at room temperature for 1 h to activate the carboxyl group of GO. Then, 100 μL cDNA (0.1, 0.5, 1.0, and 2 μM) was added to form the GO-cDNA complex due to the interaction of amino group of cDNA and the carboxyl group of GO, respectively. After reaction at room temperature for 2 h, supernatant with redundant free GO or cDNA was removed by centrifugation at 25 ℃, 10,000 rpm for 10 min. The precipitate was re-dissolved in the 100 μL 0.01 M PBS solution and incubated with 3D-rGO/AuNPs/aptamer-modified aSPCEs at 25 °C for 2 h. Finally, 3D-rGO/AuNPs/aptamer-cDNA/GO structure-modified aSPCEs were formed. The electrodes were washed with 0.01 M PBS solution and dried as with the prepared aptasensor for further use.

### 2.4. Preparation of Real Samples

The rice and oats samples were prepared according to the previous report [28]. In brief, 5 g of the rice or oat samples was mixed with 10 mL of 50% methanol in water (*v*/*v*), and an ultrasonic water bath was carried out for 20 min. The mixture was then centrifuged at 25 °C, 10,000 rpm for 10 min and the supernatant was diluted 10-fold with 0.01 M PBS (pH 7.4) for electrochemical analysis and diluted twice with 0.01 M PBS (pH 7.4) for ELISA analysis, respectively.

### 2.5. Electrochemical Measurement

Cyclic voltammetry (CV) was used to monitor the changes occurring on the modified aSPCEs surface. The CV measurement was applied in a 10 mL 0.01 M PBS solution (pH 7.4) containing 0.01 M [Fe(CN)_6_]^3−/4−^ and 0.1 M KCl from 0.6 to −0.6V at a scan rate 100 mV/s. The prepared aptasensor based on SPCEs enabled the low cost, single-use disposable POC testing of OTA. For detection, the modified aSPCEs were incubated with 10 μL OTA solution with different concentrations (0.01 pg/mL, 0.1 pg/mL, 1 pg/mL, 10 pg/mL, 100 pg/mL, 500 pg/mL, 1 ng/mL, and 2 ng/mL) in 0.01 M PBS buffer (pH 7.4) for 1 h at 37 °C, respectively. Then, OTA-reacted electrodes were rinsed with 0.01 M PBS three times. After drying at room temperature, the differential pulse voltammetry (DPV) measurements were performed under the condition of a scanning range of −0.2 V to 0.6 V and scanning rate of 0.05 V/s for OTA.

### 2.6. Apparatus

All the electrochemical measurements were conducted using a model CHI 660D electrochemical workstation (Shanghai Chenhua Instruments Co., Ltd., Shanghai, China). Disposable SPCEs with a geometrical area of 12.6 mm^2^, consisting of a carbon ink working electrode, a carbon ink counter electrode, and a silver ink pseudo-reference electrode were used as the supporting electrodes. The structure of 3D-rGO/AuNPs was observed using a transmission electron microscope (TEM) (H-7650, Hitachi, Japan). The morphology of bare and modified aSPCEs was checked with scanning electron microscopy (SEM) (Sigma 300, ZEISS, Jena, Germany). The drying of 3D-rGO/AuNPs nanocomposites under vacuum was performed with a Freeze dryer (Ningbo Shuangjia Instruments, Ningbo, China). The centrifugation was performed with a 5430R centrifuge (Eppendorf, Hamburg, Germany).

## 3. Results and Discussions

### 3.1. Principles of the Aptasensor

**Figure 1:**
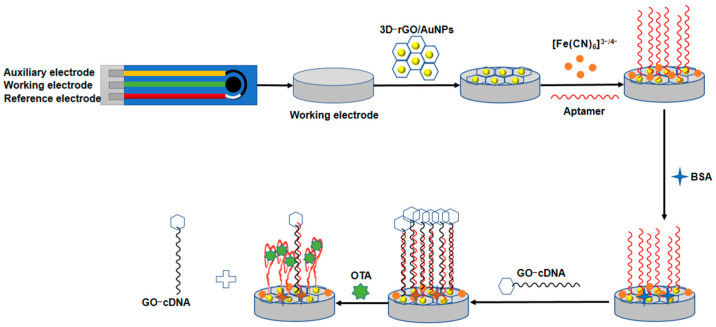
Schematic illustration of the presented aptasensor for OTA detection.

**Figure 2:**
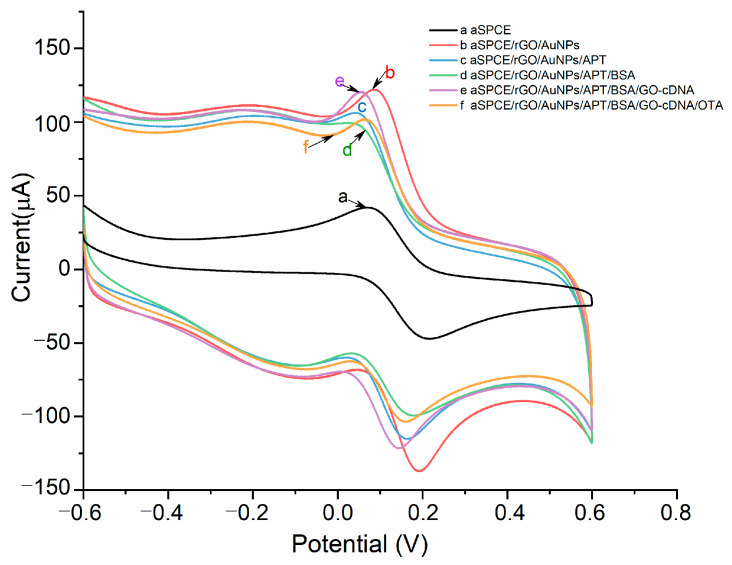
CV curves of the modified aSPCEs of every step (a, bare aSPCE; b, aSPCE/rGO/AuNPs; c, aSPCE/rGO/AuNPs/APT; d, aSPCE/rGO/AuNPs/APT/BSA; e, aSPCE/rGO/AuNPs/APT/BSA/GO-cDNA; f, aSPCE/rGO/AuNPs/APT/BSA/GO-cDNA/OTA) in a solution of 0.01 M PBS pH 7.4 containing 0.01 M [Fe (CN) _6_]^3−^/^4−^ and 0.1 M KCl.

Figure 1 shows the schematic of the presented aptasensor for OTA detection. Firstly, the 3D-rGO/AuNPs nanomaterial was modified on the aSPCEs. As can be seen from Figure 2, the peak current of curve “b” (aSPCEs/3D-rGO/AuNPs) exhibits an obvious increase compared with curve “a” (aSPCEs), indicating that the electro-conductivity of aSPCEs was improved with the 3D-rGO/AuNPs. This is due to the excellent conductive performance of rGO/AuNPs nanocomposites, facilitating the electron transfer of [Fe (CN)_6_]^3^^−^/^4^^−^ on the sensing surface. After the incubation of the SH-aptamer on 3D-rGO/AuNPs film, the peak current of curve “c” (aSPCEs/3D-rGO/AuNPs/APT) decreased because the aptamer acted as an isolating barrier to the electron transfer. The non-specific binding sites were then blocked with 1% BSA solution, in which curve “d” (aSPCEs/3D-rGO/AuNPs/APT/BSA) exhibited a small decrease in the peak current. Afterwards, the 3D-rGO/AuNPs/APT-cDNA/GO structure was formed due to the hybridization of cDNA with the aptamer when the GO-cDNA complex was added onto the electrodes. The peak current of curve “e” (aSPCEs/3D-rGO/AuNPs/APT/BSA/cDNA/GO) showed a high increase, indicating that GO-cDNA could enhance the current signal. In the presence of OTA, the specific binding of OTA to the aptamer destroyed the hybridization of cDNA and the aptamer, releasing GO-cDNA from the modified electrode. The resulting OTA-aptamer complex blocked the reaction sites on the sensing surface, leading to partial electron transfer resistance of [Fe (CN)_6_]^3^^−^/^4^^−^. In addition, the release of GO-cDNA amplified the signal change, leading to a significant decrease in the peak current as shown in curve “f” (aSPCEs/3D-rGO/AuNPs/APT/BSA/cDNA/GO/OTA).

In order to illustrate the role of GO-cDNA as a signal amplifier, the analytical performance of the proposed aptasensor compared with the 3D-rGO/AuNPs/APT-based aptasensor (without GO-cDNA, short for APT-aptasensor) and 3D-rGO/AuNPs/APT/cDNA-based aptasensor (without GO, short for cDNA-aptasensor) was investigated. The current value of ∆i_peak_ referring to the peak current change before OTA addition and after OTA addition, was used to compare the performance of the above three aptasensors. A higher current value of ∆i_peak_ indicated a better signal response of the aptasensor. For the APT-based aptasensor (without GO-cDNA), when OTA was added, the OTA could be specifically captured by the aptamer on the sensing surface. The curled complex of the OTA aptamer was formed and partly blocked the electron transfer path, leading to a decreased peak current. The ∆i_peak_ current value of the APT-based aptasensor was calculated as 3.6 ± 0.9 μA as shown in Figure S1. For the cDNA-based aptasensor (without GO), the ∆i_peak_ current value was calculated as 10.1 ± 2.1 μA, which was about 2.8-times that of the APT-based aptasensor. This result was in accordance with another report stating that a competitive strategy based on the binding of the aptamer to the OTA benefits the sensitivity of aptasensor [12]. For our proposed aptasensor, when GO-cDNA was applied, the ∆i_peak_ current value exhibited a much higher change of 17.7 ± 1.3 μA, which was 1.8-times that of the cDNA-based aptasensor, and 6-times the APT-based aptasensor, respectively. This may be attributed to the fact that GO can load the abundant cDNA, thereby enhancing the hybridization between GO-cDNA and the aptamer immobilized on the electrode surface, thus resulting in a larger current change. This result indicated that the GO-cDNA complex played a very important role as a signal amplifier for our proposed aptasensor.

### 3.2. Morphology and Structure Characterization of Modified SPCEs

**Figure 3:**
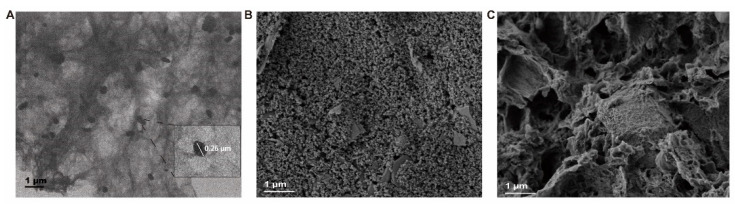
(**A**) TEM images of the prepared of 3D-rGO/AuNPs. SEM images of (**B**) the bare aSPCEs and (**C**) the 3D-rGO/AuNPs-modified aSPCEs.

TEM and SEM were used to characterize the nanostructures of 3D-rGO/AuNPs nanocomposites, and the morphology of modified aSPCEs. As is shown in Figure 3A, a large number of AuNPs with a diameter of about 260 nm were uniformly arranged on the surface of rGO, indicating the successful synthesis of 3D-rGO/AuNPs nanocomposites. Large size of AuNPs embedded in rGO can provide an efficient route for the chemisorption of the SH-aptamer during the immobilization step, leading to the sufficient binding of the aptamer on the aSPCEs surface. The SEM images of the bare aSPCEs and 3D-rGO/AuNPs-modified aSPCEs are shown in Figure 3B,C. After the incubation of 3D-rGO/AuNPs, we could see the relatively flat surface of bare aSPCEs was covered with a porous 3D-rGO structure embedded with spherical AuNPs, indicating the successful fabrication of 3D-rGO/AuNPs nanocomposites on the aSPCEs.

### 3.3. Optimization of Aptasensing Parameters

In order to achieve the best performance of the aptasensor, we optimized the most important testing parameters such as concentrations of 3D-rGO/AuNPs, aptamer/cDNA, and GO, respectively. The concentration of OTA was set as 1 ng/mL for optimization. The ∆i_peak_ current value referring to the current change between before OTA addition and after OTA addition, was used for the comparison. The higher ∆i_peak_ current value indicated better analytical performance.

**Figure 4:**
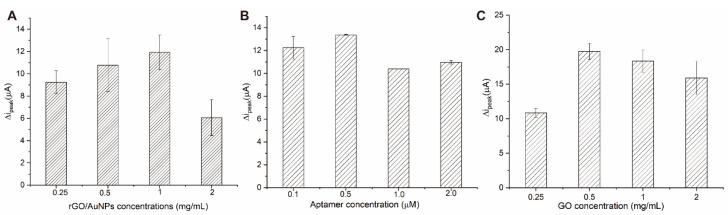
The effect of different factors: concentrations of (**A**) rGO/AuNPs; (**B**) APT; (**C**) GO on the response of electrochemical aptasensor.

The use of 3D-rGO/AuNPs nanocomposites as carriers of the aptamer can impact the analytical performance of the aptasensors. The influence of different 3D-rGO/AuNPs concentrations on the electrochemical response was investigated. It can be found from Figure 4A that the ∆i_peak_ current value increased as the concentration of the 3D-rGO/AuNPs suspension rose from 0.25 to 1 mg/mL and afterward diminished when the concentration increased to 2 mg/mL. Thus, 1 mg/mL was selected as the ideal concentration for the 3D-rGO/AuNPs suspension.

The effect of different aptamer concentrations on the performance of the electrochemical sensor was investigated subsequently (Figure 4B). With the increase in the aptamer concentration from 0.1 to 2.0 μM, the ∆i_peak_ current value reached its highest at 0.5 µM and exhibited a slight decrease after 0.5 µM, indicating a saturated capture of the aptamer on the 3D-rGO/AuNPs-modified surfaces. Therefore, 0.5 µM of the aptamer was chosen as the optimal concentration. As the normal ratio of the hybrid events between cDNA and aptamer is 1:1, it is reasonable to choose 0.5 µM as the suitable concentration of cDNA for our following experiments as well.

Finally, the influence of the GO concentration on the electrochemical response was investigated. As shown in Figure 4C, the ∆i_peak_ current value of the aptasensor increased with the rising concentration of GO and reached the highest value at 0.5 mg/mL. Following this, the ∆i_peak_ value began to decrease with the increase in the GO concentration. Hence, 0.5 mg/mL GO suspension was selected as the best concentration for the following experiments.

In conclusion, 1 mg/mL of 3D-rGO/AuNPs suspension, 0.5 µM of cDNA and the aptamer, and 0.5 mg/mL of the GO suspension were chosen as the optimal parameters of our proposed aptasensor.

### 3.4. Analytical Performance of Electrochemical Aptasensor for OTA Detection

**Figure 5:**
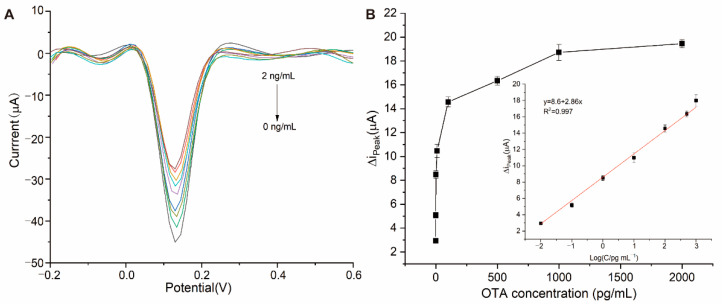
(**A**) DPV curves of aptasensor with different concentrations of OTA (0 pg/mL, 0.01 pg/mL, 0.1 pg/mL, 1 pg/mL, 10 pg/mL, 100 pg/mL, 500 pg/mL, 1 ng/mL, 2 ng/mL from lower to upper); (**B**) The trend of the ∆i_peak_ peak current with the concentration of OTA (calibration curve towards OTA detection).

Under the optimal conditions, the proposed aptasensor was incubated with different concentrations of OTA in a 0.01 M PBS solution (0 pg/mL, 0.01 pg/mL, 0.1 pg/mL, 1 pg/mL, 10 pg/mL, 100 pg/mL, 500 pg/mL, 1 ng/mL, and 2 ng/mL), respectively. As shown in Figure 5A, the DPV peak current value at 0.13 V decreased with the increasing OTA concentration, evidencing the feasibility of the proposed aptasensor. In order to investigate the analytical performance, the ∆i_peak_ current value referring to the DPV current difference between the control (0 ng/mL) and after OTA addition was calculated. As shown in Figure 5B, the ∆i_peak_ current value increased with the concentration of OTA and reached a plateau after 1 ng/mL. We found that the ∆i_peak_ current value exhibited a linear relationship with the logarithmic value of the OTA concentration from 0.01 pg/mL to 1 ng/mL. As shown in Figure 5B, the linear correlation equation can be indicated as ∆i_peak_ (µA) = 2.86 log C_OTA_ (pg/mL) + 8.6 (R^2^ = 0.997). The LOD of OTA (S/N = 3, LOD = 3 × SD/slope) which was calculated as 5 fg/mL. (Comparison of the developed disposable aptasensor with those from previous reports are summarized in Table 1)

### 3.5. Specificity and Reproducibility of the Electrochemical Aptasensor

**Figure 6:**
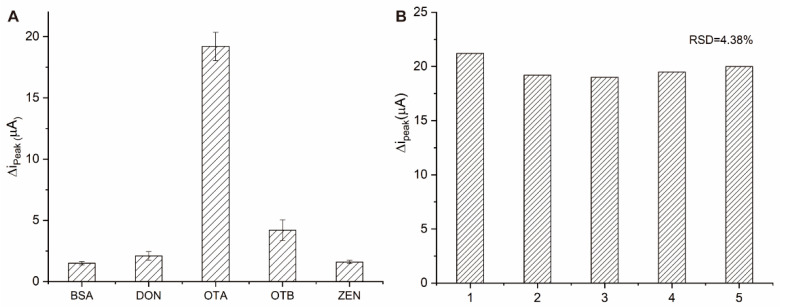
(**A**) The specificity of the proposed aptasensor against OTA, OTB, BSA, ZEN, and DON; (**B**) The reproducibility of five different electrodes.

In order to evaluate the specificity of the prepared aptasensor, different mycotoxins including OTB, ZEN, and DON, were selected as interferents. The concentration for all of the above interferents was set as 10 ng/mL while that for OTA was 1 ng/mL. As indicated by Figure 6A, the ∆i_peak_ current of OTA was significantly higher than that of the other mycotoxins. The response signal changes of ZEN and DON are negligible. The ∆i_peak_ current value of OTB is slightly higher because OTB has a similar molecular structure with OTA. Considering its 10-fold higher concentration of OTA, the specificity is thought to be acceptable. Five different electrodes were tested for the reproducibility of the aptasensor at 1 ng/mL of OTA. All the tests were performed under the same conditions and the relative standard deviation (RSD) was calculated as 4.38% (Figure 6B). In conclusion, the constructed aptasensor has satisfactory specificity and reproducibility.

### 3.6. Validation Study

**Figure 7:**
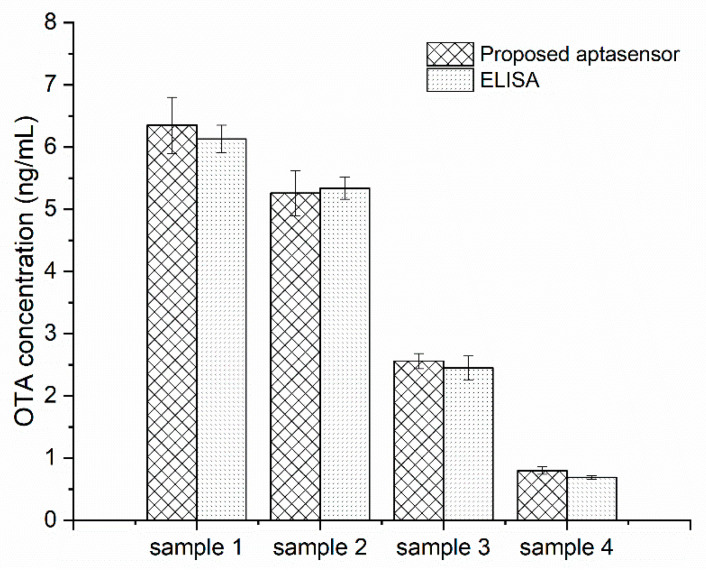
Comparison of the proposed aptasensor and ELISA for OTA detection in oat (sample 1, and 2) and rice (sample 3, and 4) samples.

In order to test the performance of the biosensor in real samples, oat (sample 1, and 2) and rice samples (sample 3, and 4) naturally contaminated with different concentrations of OTA were analyzed with the proposed aptasensor and commercial ELISA kits. The real samples were firstly treated with 50% methanol in water (*v*/*v*) under an ultrasonic water bath. The mixture was then centrifuged and the supernatant was collected as the original stock solution. It was found that the original stock solution of real samples possessed a huge matrix effect [15,31], resulting in false results. Thus, the rice and oat sample stock solutions were diluted 10-fold for accurate electrochemical analysis. According to the above calibration curve, the OTA concentration in the 10-fold solution was calculated as 0.08, 0.256, 0.526, and 0.635 ng/mL, which indicated the actual OTA concentration to be 0.8 ng/mL, 2.56 ng/mL, 5.26 ng/mL, and 6.35 ng/mL, accordingly. As shown in Figure 7, the results obtained from the two methods were in good agreement with each other, indicating the acceptable accuracy of the developed aptasensor in practical use.

## 4. Conclusions

In this work, a novel disposable electrochemical aptasensor for the ultrasensitive detection of OTA in rice and oat samples was demonstrated. Here, the competitive binding of OTA with its specific aptamer and introduction of GO-DNA was used to complete the simple signal amplification strategy. The proposed OTA aptasensors in this work exhibited quick response, convenient preparation, simple equipment, low-cost, good selectivity, high sensitivity and acceptable reproducibility. Furthermore, the aptasensor was successfully used in the detection of OTA in rice and oat samples, which was validated by ELISA. In addition, due to the ultrasensitive limit and linear range, our proposed aptasensor may be applicable to monitor the OTA exposure in human milk or urine at ultralow levels (down to 5 pg/mL) [32]. Moreover, by altering the target aptamer and cDNA, the strategy could be universally used for the detection of other mycotoxins, showing a promising potential for mycotoxins POCT in agricultural products and foods.

## Figures and Tables

**Table 1:** Comparison of the analytical performance of different disposable electrochemical aptasensors for OTA detection.

Aptasensor	Amplifier	Linear Range (ng/mL)	LOD (ng/mL)	Reference
polythiophene-3-carboxylic acid modified SPCEs	None	0.125–2.5	0.125	[19]
Disposable screen-printed Au electrodes	RecJf exonuclease and β-cyclodextrin	0.010~10	3 × 10^−3^	[20]
Thionine and IrO_2_ NPs modified SPCE	None	0.04~40	5.6 × 10^−3^	[29]
Layer-by-layer self assembly on disposable screen-printed Au electrodes	None	0.1~10	0.03	[30]
rGO-AuNPs modified SPCEs	GO/cDNA	1 × 10^−5^~1	5 × 10^−6^	This work

## Supplementary Materials

The following supporting information can be downloaded at: https://www.mdpi.com/article/10.3390/mi13060834/s1, Figure S1: Comparison of three kinds of aptasensors.
