# Supplementary figures and images for: Disposable Electrochemical Aptasensor Based on Graphene Oxide-DNA Complex as Signal Amplifier towards Ultrasensitive Detection of Ochratoxin A
Source: Micromachines (Basel). 2022 May 26;13(6):834. doi: 10.3390/mi13060834 (PMC9228252; doi:10.3390/mi13060834)

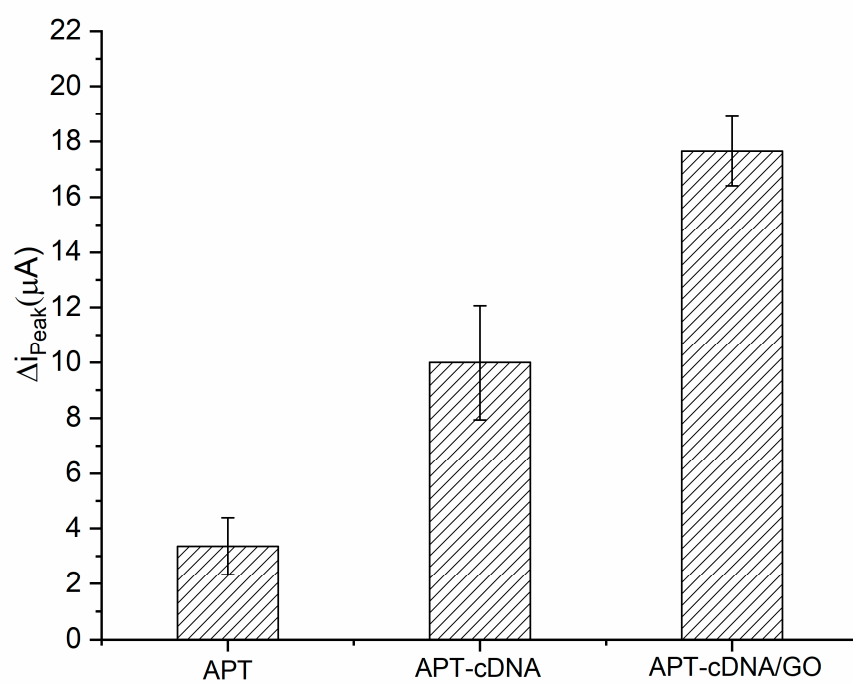

Figure S1 Comparison of three kinds of aptasensors

Supplement: Supplementary file 1 [file micromachines-13-00834-s001.zip › micromachines-1726898-supplementary.pdf]
